# COVID-19 and future pandemics: a global systems approach and relevance to SDGs

**DOI:** 10.1186/s12992-021-00711-6

**Published:** 2021-05-21

**Authors:** Tharanga Thoradeniya, Saroj Jayasinghe

**Affiliations:** 1 Department of Biochemistry and Molecular Biology, Faculty of Medicine, University of Colombo, Colombo, Sri Lanka; 2 Faculty of Medicine, University of Colombo, Kynsey Road, Colombo, 00800 Sri Lanka

**Keywords:** Covid-19, Systems, Pandemics, SDGs, Global governance, Political economy

## Abstract

**Background:**

The COVID-19 pandemic is adversely impacting modern human civilization. A global view using a systems science approach is necessary to recognize the close interactions between health of animals, humans and the environment.

**Discussion:**

A model is developed initially by describing five sequential or parallel steps on how a RNA virus emerged from animals and became a pandemic: 1. Origins in the animal kingdom; 2. Transmission to domesticated animals; 3. Inter-species transmission to humans; 4. Local epidemics; 5. Global spread towards a pandemic. The next stage identifies global level determinants from the physical environments, the biosphere and social environment that influence these steps to derive a generic conceptual model. It identifies that future pandemics are likely to emerge from ecological processes (climate change, loss of biodiversity), anthropogenic social processes (i.e. corporate interests, culture and globalization) and world population growth. Intervention would therefore require modifications or dampening these generators and prevent future periodic pandemics that would reverse human development.

Addressing issues such as poorly planned urbanization, climate change and deforestation coincide with SDGs such as sustainable cities and communities (Goal 11), climate action (Goal 13) and preserving forests and other ecosystems (Goal 15). This will be an added justification to address them as global priorities. Some determinants in the model are poorly addressed by SDGs such as the case of population pressures, cultural factors, corporate interests and globalization. The overarching process of globalization will require modifications to the structures, processes and mechanisms of global governance. The defects in global governance are arguably due to historical reasons and the neo-liberal capitalist order. This became evident especially in the aftermath of the COVID-19 when the vaccination roll-out led to violations of universal values of equity and right to life by some of the powerful and affluent nations.

**Summary:**

A systems approach leads us to a model that shows the need to tackle several factors, some of which are not adequately addressed by SDGs and require restructuring of global governance and political economy.

## Background

The Severe Acute Respiratory Syndrome Coronavirus 2 (SARS-CoV-2) causing the disease known as COVID-19 has reached a pandemic situation, temporarily changing the collective behavior of the human species and seriously affecting all other inhabitants in this planet in several ways. Lockdowns restricted movements of several populous nations affecting at least 50% of the global populations living in urban and semi-urban areas. Airlines that transport an estimated 4 billion passengers annually were grounded, industries discontinued production and globally a large proportion of the 1 billion vehicles are not on the streets. Air pollution showed a visible decrease in many cities and carbon emissions declined. With the declaration of a pandemic, the global economic activities declined, and there is increasing evidence of global economic recessions and crises [1]. Human civilization has altered its historical path, at least temporarily.

## Discussion

Conceptualizing and understanding the generation of pandemics and addressing their determinants will help inform prevention strategies against occurrence of future such global events. Of the several ways to perceive this, the authors propose a systems science approach which recognizes the close interactions between animals, humans and the environment at a global level. “One Health” is a concept, used as a global strategy to tackle the problems of Zoonoses in ways that are extended to be “holistic and transdisciplinary and incorporates multisector expertise in dealing with the health of mankind, animals, and ecosystems” [2]. Since its inception in 2008, the concept has been expanded to improve understanding of antimicrobial resistance, sustainable food systems, the development of chronic diseases and impacts of environmental pollution [3].

## Systems science

One Health is a systems approach that focuses on ecosystems, human and animal species and food systems [2, 3] while a systems science approach encompasses other components of systems such as social groupings, social structures, and socio-economic stratifications. One Health conceptualizes health to be a result of the outcomes of interactions among humans, animals and the environment. At a global level this could be viewed as interactions between subsystems within a wider holistic system, i.e. the totality of the human species, the ecosystems we live in, and the biosphere consisting of animals, plants and microbes, and physical environments.

Systems science emphasizes interactions among many more sub-systems which include social systems. These interactions are dynamic, non-linear and have multiple feedback loops, leading to emergence of novel properties. These are described as complex adaptive systems (CAS) described as “a collection of individual agents with freedom to act in ways that are not always totally predictable, and whose actions are interconnected so that one agent’s action changes the context for other agents” [4]. They exhibit features such as adaptation, lack of hierarchies, self-organization, and emergence. CAS do not have a hierarchical mode of control, and instead have multi-level ‘heterarchical’ inter-relations [4]. Thus, pathways of control flow from multiple agents and change with time. CAS also show self-organization and emergence that arise from interactions between sub-systems and the environment. An example is the emergence of complicated colony structures of termites as a result of interactions among termites. These interactions are governed by a few simple rules, and the observable outcome (the termite colony) is more than merely the sum of parts.

## Development of a generic conceptual model

**Fig. 1:**
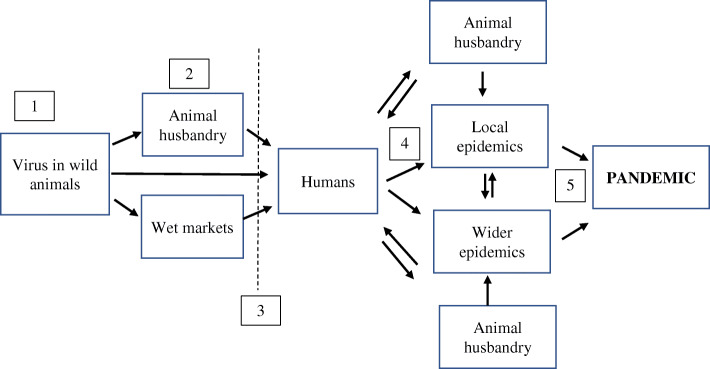
Diagram to illustrate events that lead to the Covid-19 pandemic

**Fig. 2:**
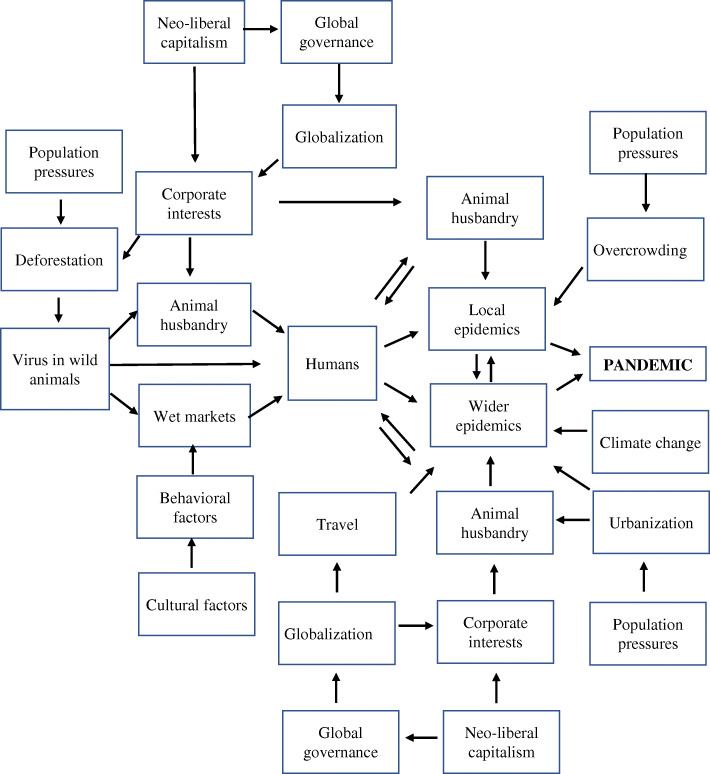
Planetary health perspective of the Covid-19 pandemic

The authors use three steps to develop the conceptual model. The first step identifies the biological events and processes from which a zoonotic virus extends to emerge as a pandemic (see Fig. 1). The second step gathers evidence that influence these biological events and processes. The evidence is mainly from diverse fields such as global health, conservation medicine, One-Health and Eco-Health [5]. In the final step, all these determinants and pathways are incorporated to the first conceptual model and linked to propose a systems models that perceives the totality of the system at a global level and describes the generation of the pandemic at a global level (see Fig. 2).

## Biological and epidemiological processes: from a virus in animals to a pandemic

The pathway of the SARS-CoV-2 originating in nature, leading to a pandemic can be viewed as several preceding events and processes (see Fig. 1):

1. Origins of virus in the animal kingdom
2. Transmission to captive and domesticated animals
3. Interspecies transmission to humans
4. Local spread and epidemics in humans
5. Global spread towards a pandemic

### Origins of the virus in the animal kingdom

A large number of emerging infections in humans originate from animals. Examples include HIV, SARS, Ebola and other infections. Of these a wide range of diseases are caused by the RNA family of coronaviruses. Theories of its origins are debated. Studies using molecular clock dating analyses of coronaviruses found common ancestor around 10,000 years ago, while recent evolutionary models have placed its origins millions of years ago [6].

The enduring relationship between bats, avian coronaviruses and their other hosts include co-divergence and co-evolution [7]. The SARS-CoV (that caused SARS) emerged from Chinese *Rhinolophus affinis* (horseshoe bats) from a milieu of several similar viruses that existed in this species of bats [8]. The corona viruses were also isolated from clinically normal wild animal species (e.g. Himalayan palm civets and raccoon dog) in wild animal markets in Shenzen, the People’s Republic of China [9]. Thus, reservoirs of the corona viruses are circulating among different species, some asymptomatic and a few causing symptomatic illness though bats form their main reservoir [10]. The novel SARS-CoV-2 is the seventh corona virus known to infect humans, emerged from this milieu and it is found in its natural host the bat *Rhinolophus affinis* or *Manis javanica* (Malayan pangolins) illegally imported into Guangdong province. Modeling and simulation studies indicate that “over two-thirds of SARS-CoV-2-like zoonotic events would be self-limited, dying out without igniting a pandemic” [11].

### Transmission to domesticated animals

Transmission of corona viruses across species is well known. The recent report of the Joint WHO-China Study on the origins of COVID-19 found the most closely related forms of the virus in bats and pangolins [10]. However, they were not sufficiently similar to be the direct link suggesting the need to search for alternative reservoirs. Cats and mink are highly susceptible to the virus and are considered potential reservoirs.

The new host could act as conduits of transmission rather than as reservoirs. Civets were favored as intermediate hosts in the previous epidemic of severe acute respiratory syndrome (SARS) and pangolins in the case of COVID-19. However, epidemiological studies in humans and similar tracing of infections in animals have failed to find an intermediate source.

Similar situations when new CoV strains emerged from mutation of existing strains or changed its virulence from existing strains have been observed in the past, e.g. the emergence of a new group I porcine CoV responsible for the porcine epidemic diarrhoea CoV (PEDV) in 1970s and 1980s [12]. A corona virus in bats, which was non-pathogenic to bats was implicated in causing fatal diarrhoea in swines [13]. They also jumped species to infect farm animals (e.g. turkey and chicken) and domestic pests such as rodents [14–16]. Other species affected by novel mammalian coronaviruses include camels, bats, mice, dogs, and cats [17]. The resultant illness of often mild to severe with enteric or respiratory symptoms or a more systemic febrile illness.

Although there are a few reports of SARS-Cov-2 transmission from humans to animals there is little evidence that domestic animals are an important reservoir of SARS-Cov-2 virus. A comprehensive understanding of the virus spread from human to animal and *vise versa* is crucial for its control and prevention of future similar events. As such, this pathway is included when developing the generic conceptual model.

### Transmission to humans

The ability of the Covid-19 to spread is its mechanism of replication and mutation, common to many RNA viruses [18]. RNA viruses have high rates of error during replication due to substitutions, insertions and deletions [19]. This makes it an excellent candidate for rapid host switching, and the emergence of variants that could have properties of higher transmissibility or pathogenicity. The mutation that enabled a species jump is likely to be localized in its ability to invade the cells of another animal species or humans. Evidence supports the view that ancestors of SARS-CoV in bats developed the capacity to enter human cells only after it mutated to produce a viral glycoprotein that binds to angiotensin converting enzyme 2 (ACE2) receptors on cell surfaces [20].

Increasing contact between animals and humans augments the risks of inter-species transmission from wild or domestic animals to humans, and there are three such situations: extension of human settlements closer to forests, wet markets and animal husbandry.

#### Extension of human settlements

At the global level, the extents of deforestation is a proxy measure of extension of human settlements. From 1990 to 2015, the global forest cover has reduced from 31.6% of the earth’s land surface area to 30.6% [21]. Almost 50% of this is due to farming, grazing of livestock, mining for metals, gas and fossil fuels. These, especially the former two activities promote interactions between wild animals and domesticated animals and thereby humans. The reasons and rates of deforestation vary: in Malaysia and Indonesia its due to plantations for palm oil and in the Amazon, it’s for cattle ranches, farms, and soya plantations [22]. In addition, in Asia and Africa expanding population and limited land has led to human settlements within and near to forests, progressively forcing wild animals into closer contact with humans and domesticated animals.

#### Wet markets

In wet markets, different live species (vertebrates and invertebrates) are caged close to each other in busy urban settings. China wet markets are implicated in the Covid-19 and are now considered an important source of its spread across species and to domestic animals and humans [23]. The presence of wet markets in China, Vietnam and Taiwan is a cultural preference of ‘freshness’ as an important aspect of culinary appreciation. The environment and appearance of wet markets (liberal use of water and wetness) give a sense of freshness which is resonant with their culinary culture. This is reinforced by the ambiance, the sensory cues such as free use of water and wetness, and trust created by vendors to provide fresh food. Wet markets were a source of H5N1 avian influenza in humans and probably the current Covid-19 pandemic.

#### Animal husbandry

Factory farms contain large population of domesticated animals which form an important reservoir for any potential infections. Though yet to be recognized as a major factor in COVID-19, there is evidence of human spread from farmed minks (*Neovison vison*) in the Netherlands [24]. Surveillance data showed large transmission clusters of SARS-CoV-2 with increased mortality in minks and subsequent evidence of SARS-CoV-2 infection with an animal sequence signature in 68% of farm residents, and employees. This led to culling of millions of minks in Netherlands and Denmark, similar to what was done in Europe during the epidemics of 2009 (H1N1) pandemics when billions of farm animals were destroyed.

### Local spread and epidemics

The situation to generate an outbreak is achieved when human-to-human transmission becomes possible and a critical mass of humans are infected. The local spread begins to propagate when the basic average reproductive number exceeds unity [25]. More detailed analyses have shown the importance of super-spreading events in generating the epidemic [26]. There is evidence of the presence of multiple small epidemics in cities prior to the eruption of a major epidemic [27] Genome sequencing from Washington state indicates that there was cryptic transmission of the COVID-19 from a single source as early as January 2020.

Overcrowding promotes the spread of epidemics through aerosols, droplets and fomites and studies from China show that the epidemics have larger total attack rates and last longer than less dense and less populated cities [28]. Population pressures have exacerbated the process of urbanization and the congestion within cities. Key determinants of congested cities are rapid urbanization coupled with an increasing population. The current global population of 7.8 billion is the highest ever number of human inhabitants in the planet and it continues to increase. Increased human mobility within cities and countries are other factors promoting epidemics and the interventions restricting mobility are well known to control the spread of the disease [29]. There is research to show that connectivity within cities is likely more important for the the spread of COVID-19 than population density [30].

Unplanned urban developments with its combination of overcrowding, poor levels of personal hygiene due to scarcities in amenities such as running water, inadequate sanitation, confined poorly ventilated houses, production of solid waste and high air pollution are all likely to accelerate the spread of COVID-19 and other similar respiratory diseases within and across households. The detection of the COVID-19 RNA in solid waste feces suggest another potential pathway of spread of the virus in urban and peri-urban areas [31].

On the other hand, non-communicable diseases that are linked to unhealthy diets and lifestyles are main consequences of urbanization. These are associated with more severe illness and higher mortality with Covid-19.

### Global spread towards a pandemic

Global spread is mainly due on international travel, especially during the incubation period [32]. In recent times humans have become increasingly mobile and travel to almost all parts of the planet. It is estimated that 4.5 billion passengers were carried by airlines in 2019, giving an opportunity for rapid and wide spread of infections and to generate pandemics [33].

In the case of COVID-19 the major spread has been infected humans who travelled to different locations of the globe as tourists or for work. Human species are mobile in relation to individual distance covered as well as frequency of movement. The persistence of the virus on plastic and metal surfaces, detection among baggage handlers in airports, spread within the cabins of airlines and cruise ships all indicate how its spread across borders was facilitated by non-human ‘artefacts’ [34].

Another factor is the possibility of particles in air pollution and dust storms acting as careers of viruses or worsening the severity of illness, thereby promoting virus excretion and its spread [35].

The direct role of climate change on generating the COVID-19 local epidemics is less well known. In the case of influenza, a warming climate, the rapid weather variability intensifies the epidemic risk that may increase even by 50% in some northern mid-latitude regions [36]. Researchers in China found that mean ambient temperature had an impact in low temperature (below a mean of 3^0^ C) [37]. This could be partly a direct effect of ambient temperature (and humidity in the case of influenza) on survival of the virus in the environment or indirect effects that lead to changes in human behaviours, e.g. use of heaters or air conditioners during warmer weather which increases circulation of air within a confined space.

Climate change undoubtedly exert pressure on wildlife through which it initiates the various indirect mechanisms paving way to extinction. A healthy eco-system with biodiversity could contain viruses and prevent them from jumping to new host species from the wild. Viruses emerge and fade without much damage to its wild hosts in undisrupted, diverse eco-systems which are well separated from human inhabitants. Thousands of mammalian viruses may exist potentially harmful to humans and domesticated animals. Urgent actions leading to biodiversity protection are imperative for prevention of climate change as well as the next pandemic. It is well documented that almost half of zoonoses emerged after 1940 could be traced to disrupted biodiversity [38].

Though yet to be recognized as a major factor in COVID-19.

Domesticated animals are fertile grounds for potential global epidemics [39]. The large-scale outbreaks of SARS-CoV-2 in mink farms were initially reported from Denmark and Netherlands [24], followed by other parts of Europe, Canada and the United States leading to a de facto shutdown of the mink industry in these countries [40].

There is yet no evidence of large scale human COVID-19 epidemics traced to factory farming, transmission by ingesting infected materials or animal husbandry. However, whole-genome sequencing confirmed mink-to-human transmission of SARS-CoV-2 in clusters of employees and their contacts in mink farms in Netherlands [24]. Further investigations also indicated unique mutations in SARS-CoV-2 variants, existence of multiple generations of the virus as well as a faster evolutionary rate of the virus in minks, leading to concerns on effectiveness of the current vaccines.

These outbreaks are of grave concern because of the magnitude of global factory farming industry. An estimated 70 billion animals are bred in farms for human consumption and factory farms house millions of poultry, pigs, cattle and other animals in constrained and controlled environments. If these animals and their meat products act as vectors, the infection could potentially spread locally as well as globally [41]. In the past several such epidemics have been reported, and examples include, Bovine Spongiform Encephalopathy caused by prions and spread by infected beef products that have nerve tissues, salmonella through chicken eggs, and Middle East Respiratory Syndrome (MERS) through direct or indirect contact with infected dromedary camels [42].

## A systems science perspective and SDGs

The basic structure of Fig. 1 was developed further by incorporating the factors that were described in the previous section and identified so as to promote global spread of COVID-19 (see Fig. 2). They constitute more global level systems factors.

From a systems science perspective, the COVID-19 pandemic could be viewed as an emergent property of a complex dynamic system at the level of the whole human species and planet. The components of the system include biological properties of the virus, ecological factors including biodiversity, human and animal behaviors, and demographic changes. Global actions on these determinants are essential to prevent future pandemics because there are drivers at a global level. Global interventions would therefore be required to modify or dampen these generators if we are to prevent future periodic pandemics, preserve human and animal health, and sustain the planet. The model suggests several pathways that could have contributed to generating the current COVID-19 pandemic and proposes the possibility of future pandemics emerging from these global level determinants.

Those identified include ecological processes (e.g. climate change affecting the planet), civilizational (i.e. values and social structures that promote urbanization, lifestyles and corporate interests), physical (e.g. increased mobility of the species and its artefacts, human activities such as deforestation, improper waste management) and demographic (unprecedented growth in the numbers of the human species).

## Preventing COVID pandemics and SDGs

We now relate the factors identified in the model (Fig. 2) and to the SDGs. This will enable us to identify strategies needed to prevent future pandemics, over and above SDGs.

Perusing the literature, there are areas of overlap between the factors identified in the model and almost all the SDGs, especially those in relation to urbanization with overcrowding (Goal 11), climate change (Goal 13) and deforestation (Goal 15). Each of these Goals in turn are linked to other targets and goals [43–45].

### Urbanization with overcrowding

Urbanization is one consequence of population growth, while concentration of economic activities and availability of services attracts more people towards it. Cities already constitute above 55% of the population and contribute to almost 80% of global economic growth [46].

The growing population and migration lead to rapid and unplanned urbanization.

SDG 11 is to “make cities and human settlements inclusive, safe, resilient and sustainable”. Almost 60% of COVID-19 have been in urban areas thus highlighting the importance of cities in generating or accelerating the pandemic [47]. In order to make healthy sustainable cities for the future, urbanization must be linked to affordable, reliable, sustainable and modern forms of energy production (i.e. SDG Goal 7), sustainable consumptions and production patterns (SDG 12) and combat climate change and its impacts (SDG 13) [44, 45].

### Climate change

SDG 13 is to “take urgent action to combat climate change and its impacts” in order to counter rapid rates of global warming, emission of green-house gases and occurrences of natural disasters. Goal 13 and many others are linked such as Goal 7 (appropriate energy productions), Goal 9 (reliable and sustainable industry, innovations and infrastructure that is accessible equitably), making settlements inclusive, Goal 11 (safe, resilient and sustainable cities), Goal 12 (having sustainable consumptions and production patterns) and Goal 12 (combating climate change and its impacts) [44, 45].

### Deforestation

This is explicitly considered as SDG 15 which states: “Protect, restore and promote sustainable use of terrestrial ecosystems, sustainably manage forests, combat desertification, and halt and reverse land degradation and halt biodiversity loss”. The Sustainable Development Goals Report 2020 has identified “deforestation, and habitat encroachment are primary pathways of transmission for emerging infectious diseases, including COVID-19” [40]. Deforestation is partly driven by poorly sustainable agricultural systems that are striving to ensure food security. This is dealt by Goal 2 which is to “End hunger, achieve food security and improved nutrition and promote sustainable agriculture” [44, 45].

## Factors less well addressed by SDGs

The model identifies other factors that are less well addressed by SDGs: population pressures, culture-based behavioral factors, and process of rapid globalization, corporate interests, extensive travel / transport.

### Population pressures

The SDGs do not specify targets for population growth. However, there are several studies on the adverse impact of population on biodiversity, bio-gas-chemical cycles, land use and pollution levels [48]. The rapidity in the rate of growth of human populations has contributed substantially to the loss of biological diversity and deforestations on a mass scale [49]. Population growth and associated expansion of their habitats have played key roles in driving species to extinction. During the last 100 years, the Earth has lost 400 vertebrate species which is 1000 times faster than the normal course of evolution [50].

Female empowerment, access to reproductive health and gender equity are all factors that would reduce the population explosion that would be fermenting grounds for future pandemics.

### Cultural and behavioral factors

There is hardly a mention about culture-based behavioral factors in SDGs. Though financial incentives play a key role, several local and international food habits are determined by cultural factors. Countering the popularity of wet markets with its live or recently killed animals will require promoting a new culture and alternative behavioral patterns that would promote demand for freshly prepared, yet well-packaged food. These actions have to be combined with strict monitoring of hygienic standards, legislations and enforcement to improve hygiene practices and prevent sales illegal wild animal meats [51].

The scenario becomes more complex when cultural factors influence the actions taken to mitigate outbreaks in animals, such as with the farmed minks leading to plans cull and a de factor closure of mink industry in some countries in Europe. Plans to cull of millions of animals often lead to conflicts with animal welfare activists. Their demands to phase-out a cruel industry resonates with a food culture that is less dependent on eating and enjoying animal flesh.

#### Globalization, corporate interests, travel / transport across vast geographic space

The process of globalization is facilitated, encouraged and promoted by corporate interests that trade across borders in order to maximize profits and exploit natural resources from different regions of the globe. One study linking commodities and their supply chains found that 30% of extinction threats were due to international trade [52].

The current wave of globalization is driven by factors such as economic and financial, political, technological and social factors such as culture, mass media, and values of consumerism [53].

Economic and financial factors that promote globalization are lower trade and investment barriers by nations and expansion of the financial sector. Political factors relate to government policies that facilitate trade across borders (e.g. promotion of foreign direct investment). Technology has accelerated globalization by developing low-cost, widespread, and rapid communication technologies (e.g. the internet) and enabling rapid distribution of products (e.g. multinational food chains through container shipments). Though beneficial from some perspectives, they could leave large ecological footprints. For example, food supply chains that span across continents could emit substantial pollution, require burning of fossil fuels and lead to unsustainable consumption that are unsustainable. These require action through novel concepts such as sustainable growth, circular economies and recycling of waste. Social factors such as mass media have brought a degree of cultural dominance and convergence, and promotion of travels across borders and the spread of values such as consumerism.

The economic, financial and political factors that drive globalization are biased towards corporate interests and are influenced by the structures, processes and mechanisms of global governance which reinforce asymmetries in power between the affluent and poorer nations [54].

These are rooted in historical power structures and ideologies, for example histories of British colonization that have led to the formation of a Commonwealth of Nations and the Second World War that led to a UN Security Council that has a self-appointed group with powers to veto over any resolutions. These power structures may have fueled the widening of inequities or at the least, failed to counter the formation of elites or super-rich at a global level. For example, they were impotent and passive observers while the richest 22 individuals accumulated wealth more than the combined wealth of all women in Africa! [55].

The Lancet’s Commission on Global Governance for Health identified five issues perpetuating this situation: insufficient representation of actors such as civil society in decision-making processes; weak accountability mechanisms; procedures that sustain existing disparities in power and maldistribution of health; inadequate means to protect health in non-health policy-making arenas; absence of institutions to protect and promote health [54].

They proposed a way forward though a policy forum to frame and debate policies that impact on health and health equity, establishing health equity impact assessments within international organizations, strengthening human rights instruments for health, committing to global solidarity beyond traditional development assistance, and acknowledging the need for global cross-sectoral action and justice to address health inequity. However, 6 years after the Commission’s call for action, the failures global governance was revealed in the maldistribution of COVID vaccines [56].

The very nations that preached social justice and fair play in international forums (e.g. Canada and members of the European Union) did the exact opposite by monopolizing the markets, purchasing vast stocks, and hoarding vaccines well above their immediate needs. Such violations of trade rules and discrimination that deprived many nations of a steady basic supply, were crouched behind terms such as ‘vaccine nationalism’. A recent Lancet editorial lamented of a “……startling lack of solidarity between countries”….. and complained that “Rich nations have given money to COVAX and paid lip service to the idea of vaccines for all while scrambling to buy up all the doses they can” Perhaps these actions should be labeled as a scandalous violation of our basic values at a time of a pandemic. It eroded values of treating all humans in the planet as equal and having a right to life, irrespective of the international borders that confine them to nation-states.

There are concerns whether it is ever possible to reform such ingrained and historically determined politico-economic structures that are a form of neo-liberal capitalism. It is difficult to expect them to spontaneously evolve to become fairer and more equitable.

Hopefully, a transformative approach such as the Sustainable Development Goals would achieve these noble objectives through popular consensus, democratic actions and non-violent means [57, 58]. However, the already fragile initiative appears to have been compromised since the COVID-19 pandemic that has devastated economies, destroyed livelihoods, and continues to crush communities [59]. As a result, there are calls for recalibrating the targets e.g. school closured during the pandemic have reversed achievements in getting more children to primary schools. There is a more radical proposal to overhaul SDGs by decoupling goals from economic growth. This is because economic growth has failed to yield adequate benefits to a majority while absorbing large volumes of subsidies to industries that compromise many SDGs. An example is that “Each year, citizens are paying the equivalent of the gross domestic product of Japan to prop up an industry (*i.e. the fossil fuel industry*) that is among the principal causes of climate change and unsustainable development. This money should be spent on achieving the goals, not undermining them” [59].

## Conclusions

We have used a systems approach to understand the COVID-19 pandemic and to develop a model which recognizes the close interactions between health and wellbeing of animals, humans and the environment. The proposed model suggests that the COVID-19 pandemic and future pandemics are likely to emerge from ecological processes such as climate change, loss of biodiversity, anthropogenic social processes (e.g. corporate interests, culture and globalization) and world population growth. Intervention would therefore require modifications or dampening these generators. It provides a framework for policymakers and informed members of civil society to grasp and systemic nature of the determinants that have triggered a pandemic. Addressing some of these determinants coincide with SDGs such as sustainable cities and communities (Goal 11), climate action (Goal 13) and preserving forests and other ecosystems (Goal 15) further justifying the need to accelerate achieving them. Perusing the literature, there are areas of overlap between the factors identified in the model and almost all the SDGs, especially each of these Goals in turn are linked to other targets and goals.

Furthermore, the model highlights the need to address certain determinants such as corporate power, global governance and excess population growth, that are not directly mentioned in the targets of SDGs.

## Data Availability

None.
